# Association of Antibiotic Usage With Food Protein-Induced Enterocolitis Syndrome Development From a Caregiver’s Survey

**DOI:** 10.1097/PG9.0000000000000132

**Published:** 2021-10-25

**Authors:** Jeanelle Boyer, Lizzy Sgambelluri, Qian Yuan

**Affiliations:** From the * Keene State College, Keene, NH; † MassGeneral Hospital for Children, Boston, MA.

**Keywords:** food protein-induced enterocolitis syndrome, FPIES, acute FPIES

## Abstract

Food protein-induced enterocolitis syndrome (FPIES) is a frequently misdiagnosed, serious, non-IgE–mediated food allergy, and the precise mechanism of disease is unknown. Acute FPIES typically presents with repetitive, profuse vomiting approximately 1 to 4 hours post-ingestion of a food trigger. Chronic FPIES is considered less common and less well characterized.

**Objective:**

We aimed to better describe FPIES and identify factors that may influence FPIES development through use of a self-reported, caregiver’s survey.

**Methods:**

FPIES and allergy-free infant caregivers completed a survey regarding lifestyle factors that may influence allergy acquisition such as antibiotic usage and delivery mode. FPIES caregivers reported symptoms, number of food triggers, type of FPIES, and symptoms from breastmilk ingestion. FPIES infants were compared to allergy-free infants to identify factors potentially associated with FPIES.

**Results:**

Infant and prenatal maternal antibiotic usage was higher in FPIES infants compared to allergy-free infants (43.8% versus 20.6% and 48.8% versus 23.57%, respectively; *P* < 0.05). When compared to infants with acute FPIES alone, infants described as both acute and chronic FPIES reported earlier onset of symptoms, more nonspecific symptoms, and symptoms triggered by breast milk, more antibiotic exposure, and more food triggers (*P* < 0.05).

**Conclusion:**

Antibiotic usage was significantly higher in FPIES infants when compared to allergy-free infants. Work is needed to elucidate the role of antibiotic usage in the etiology of FPIES. Infants reported to have both acute and chronic FPIES were significantly different from infants with acute FPIES alone, highlighting the need to more closely examine these different subtypes of FPIES.

**What Is Known**

- Food protein-induced enterocolitis syndrome (FPIES) is a frequently misdiagnosed, severe, non-IgE–mediated allergy affecting infants and children that can cause profuse vomiting and potentially shock.
- The cause of FPIES is yet unknown, and due to misdiagnosis, treatment is often delayed.

**What Is New**

- Prenatal and infant antibiotic usage was more prevalent in FPIES infants when compared to allergy-free infants.
- Infants with both acute and chronic FPIES had earlier onset of symptoms, were more likely to react to breast milk, had greater antibiotic exposure, and more food triggers when compared to infants with just acute FPIES.

Food protein-induced enterocolitis syndrome (FPIES) is a frequently misdiagnosed, non-IgE–mediated food allergy that primarily affects infants and young children. Acute FPIES reactions typically present with repetitive vomiting, lethargy, pallor, and sometimes diarrhea approximately 1 to 4 hours after consuming the allergen. If the reaction is severe and hypotension leading to shock develops, it could be life-threatening. Chronic FPIES is less clearly defined and has been characterized as chronic vomiting or diarrhea, potentially with failure to thrive with ongoing exposure to a trigger food.^1^ Currently, the diagnosis of FPIES is frequently delayed due to a widespread lack of recognition of the disease. One survey indicated that 80% of pediatricians had little-to-no knowledge of FPIES.^2^ These challenges often lead to misdiagnosis, unnecessary medical testing, frequent hospitalizations for acute reactions that were thought to be viral, and overall increased stress for FPIES families.^2^ Diagnosis is strictly clinical, and allergy testing, such as skin prick testing and specific IgE, is usually negative. While FPIES was formally recognized in the 1970s, it did not receive a diagnostic code until October of 2015.^1^ Epidemiological data are still lacking, making it difficult to estimate the prevalence of FPIES in the United States. A recent population-based survey of 38 408 children estimated the FPIES prevalence to be 0.5% in the United States.^3^ A birth cohort study of 158 100 US infants found an incidence between 0.17% and 0.42%.^4^ In an Australian study, the estimated annual incidence of FPIES was 15.4 per 100 000 children younger than 2 years.^5^ In a more recent Spanish birth cohort study, a cumulative incidence of FPIES was 0.7%.^6^

There is limited research exploring environmental and lifestyle factors that could influence the development of FPIES. Early life factors, such as mode of delivery and breastfeeding, impact the gut microbiome,^7^ and the factors that influence childhood exposure to microbes have been found to influence IgE-mediated allergic disease.^8^ The maternal microbiome may also play a significant role in infant immune system development and allergy acquisition,^9^ and evidence suggests that maternal antibiotic usage may influence IgE-mediated allergy and asthma development in offspring.^10,11^ At this time, research into non-IgE allergy and the microbiome is very limited, with some associations seen between the esophageal microbiome and eosinophilic esophagitis.^12^

The primary aim of this study was to examine factors, such as the rate of antibiotic usage, breastfeeding, and C-section, for their association with FPIES by comparing survey responses from FPIES infant caregivers to allergy-free infant caregivers. The second aim was to describe symptoms of FPIES and other nonspecific symptoms in infants with FPIES from the caregiver’s perspective. Parents of FPIES children still struggle to receive a timely diagnosis due to general lack of knowledge about FPIES. In-office oral food challenge, the gold standard for FPIES diagnosis, may be impractical and carries some risk. Many times, diagnosis is based upon the caregiver’s description of their child’s history. Better awareness of the caregiver perspective may increase recognition of FPIES by medical providers and reduce time to diagnosis.

## METHODS

Parents or guardians of allergy-free infants or infants with physician-diagnosed FPIES, aged 12 months and younger, completed an online, self-reported, cross-sectional survey delivered by Qualtrics (https://www.qualtrics.com/research-core/survey-software/, RRID:SCR_016728). The survey was open from April 2016 until September 2016, and participants were recruited primarily through social media and press release. The majority of FPIES participants were recruited through postings by the FPIES Foundation on social media, as well as from an online FPIES Support group on Facebook. The FPIES support group is an active group with over 10 000 members and is described as a support forum for families caring for a loved one diagnosed with FPIES. The majority of the allergy-free participants were recruited through a press release. The study was approved by the Keene State College Institutional Review Board, and written, informed consent was obtained by all parents and legal guardians before completing the survey. Allergy-free infants were free of allergy and allergy-related disease, including FPIES, IgE-mediated allergy, eczema, asthma, and other non-IgE–mediated allergies, at the time of survey. FPIES status was self-reported by the caregivers and not confirmed by a study physician. However, before enrollment, participants were screened to ensure that they had received a physician-confirmed diagnosis of FPIES before participation. All caregivers of infants with FPIES stated that their child had received a physician’s diagnosis of FPIES and confirmed this for a second time in the completed survey. Type of FPIES was also self-reported. Participants were asked to state whether or not their infant had acute FPIES, chronic FPIES, or both acute and chronic FPIES. Type of FPIES was defined in the survey question as follows: “acute FPIES is characterized by an episode of sudden onset vomiting approximately 2-4 hours after ingesting a food trigger. The acute vomiting may be associated with lethargy, pale skin, cold and clammy skin, and potentially shock. Chronic FPIES is typically characterized by chronic diarrhea (may or may not contain mucus/blood), chronic abdominal pain, intermittent vomiting without shock like symptoms and potentially difficulty gaining or maintaining weight.”

Self-reported lifestyle and demographic information included age, gender, maternal and infant antibiotic usage, delivery mode (C-section or vaginal), feeding method (breastfeeding, formula, or combination of both), and the presence of a family pet. Participants also responded to survey questions that addressed type of FPIES symptoms, number of FPIES food triggers, age of symptom onset, and symptoms thought to be secondary to maternal diet through breast feeding.

A series of χ^2^ analyses and contingency tables were utilized to test differences between expected and observed values and to evaluate associations between 2 or more categorical variables of interest. Count served as the numerical response variable. Five predictor variables were included in the original model: FPIES (binary; yes/no), condition (none, acute, chronic, both acute and chronic), gender, antibiotic use (binary; yes/no), and timing at which antibiotic usage occurred. Significance was tested at the 95% confidence interval (α = 0.05). Since clinical guidelines for chronic FPIES diagnosis are lacking, further statistical analysis was completed, excluding those that reported only chronic FPIES. In this model, χ^2^ analysis compared the antibiotic usage between infants with acute FPIES and both acute and chronic FPIES to allergy-free infants. Further χ^2^ analyses were used to compare the age of onset, rate of breast milk reactivity, antibiotic usage, symptoms, and number of food triggers between infants with just acute FPIES and infants with both acute and chronic FPIES. Analyses were performed to verify data that met all test assumptions before model building and testing.

Model construction and analysis was performed using EMT,^13^ vcd,^14^ ggplot2,^15^ plyr,^16^ and dplyr^16^ packages in R statistical computing software (http://www.r-project.org/, RRID:SCR_001905).

## RESULTS

Ninety-one participants enrolled in the study and 75 completed the survey, including 41 caregivers of FPIES infants and 34 caregivers of allergy-free infants. Seventy-one participants were from the United States, 3 participants were from Canada, and 1 participant was from Greece. At the time of the survey, the mean FPIES infant age was significantly greater than the mean allergy-free infant age (10 versus 8 months, respectively; *P* = 0.00001). At 9 months post-survey, follow-up with allergy-free infants revealed no reported change to their allergy status at that time.

Of the 41 FPIES participants, 23 reported both acute and chronic types of FPIES reactions, 8 reported chronic FPIES, and 10 reported acute FPIES (Table 1). In selecting both acute and chronic or chronic FPIES on the survey, the participants agreed that they had symptoms matching the chronic diagnosis (definition provided on the survey). However, due to a lack of well-defined clinical guidelines for chronic FPIES diagnosis, respondents reporting chronic FPIES were considered presumptive. The survey did not ask participants to distinguish the type of reaction/symptoms per food, so it is not clear as to whether or not these reactions were to the same or different foods.

**TABLE 1.** Self-reported characteristics of FPIES respondents

Characteristics	No. of FPIES infants, %
Total FPIES infants	41 (100%)
Type of FPIES	
Acute	8 (19.5%)
Chronic	10 (24.4%)
Both acute and chronic	23 (56.1%)
Age of onset: all FPIES	
Birth	5 (12.2%)
0–2 months	20 (48.8%)
3–4 months	3 (7.3%)
5–6 months	10 (24.4%)
7–8 months	2 (4.9%)
9–12 months	1 (2.4%)
Reaction symptoms: all FPIES	
FPIES-specific symptoms	
Vomiting	36 (87.8%)
Diarrhea without blood or mucous	30 (73.2%)
Diarrhea with blood or mucous	23 (56.1%)
Lethargy	29 (70.7%)
Weight loss	17 (41.5%)
Failure to thrive	9 (22.0%)
Shock	9 (22.0%)
Nonspecific symptoms	
Acid reflux	32 (78.0%)
Increased night waking	31 (75.5%)
Changes in mood/temperament	30 (73.2%)
Abdominal pain	28 (68.3%)
Increased gas	25 (60.1%)
Rash	22 (53.7%)
Constipation	18 (43.9%)
Dehydration	14 (34.1%)
No. of food triggers: all FPIES	
1	3 (7.3%)
2	4 (9.8%)
3–4	13 (31.7%)
5–6	3 (7.3%)
7+	18 (43.9%)
Symptoms in response to breastmilk: all FPIES	34 (82.9%)
FPIES-specific symptoms	
Diarrhea with mucous or blood	24 (58.5%)
Diarrhea without mucous or blood	16 (34.1%)
Vomiting	13 (31.7%)
Weight loss	5 (12.2%)
Failure to thrive	5 (12.2%)
Lethargy	4 (9.8%)
Shock	1 (2.4%)
Nonspecific symptoms	
Acid reflux	27 (65.9%)
Skin rash	19 (46.3%)
Increased night waking	24 (58.5%)
Abdominal pain	23 (56.1%)
Increased gas	22 (53.7%)
Changes in mood or temperament	20 (48.8%)
Constipation	3 (7.3%)
Dehydration	1 (2.4%)
Other allergic condition: all FPIES	
IgE-mediated food allergy	7 (17.1%)
Atopic dermatitis	3 (7.3%)
Anaphylaxis	1 (2.4%)

FPIES = food protein-induced enterocolitis syndrome.

The most common age ranges for symptom onset were 0 to 2 months (48.8%) and 5 to 6 months (24.4%, Table 1). FPIES participants reported a wide variety of symptoms that were further grouped into “FPIES-Specific Symptoms” and “Non-Specific Symptoms” based upon the recent clinical diagnostic guidelines (Table 1).^1^ FPIES-specific symptoms included vomiting, diarrhea with blood or mucous, diarrhea without blood or mucous, lethargy, weight loss, failure to thrive, and shock. Nonspecific symptoms included acid reflux, increased night waking, changes in mood, abdominal pain, rash, constipation, and dehydration.

The vast majority of FPIES participants reported vomiting (87.8%) as a reaction symptom. Other commonly reported FPIES-specific symptoms included diarrhea (73.2%), lethargy (70.7%), and diarrhea with blood or mucous (56.1%). Less commonly reported were two more serious symptoms of FPIES: failure to thrive (22%) and shock (22%). Over half of the participants also noted the following nonspecific symptoms: changes in mood/temperament, abdominal pain, increased gas, acid reflux, increased night waking, and skin rash. The majority of respondents reported multiple food triggers with 43.9% reporting 7+ triggers, 7.3% reporting 5 to 6 triggers, and 31.7% reporting 3 to 4 triggers (Table 1). In total, 87.8% of all FPIES infants reported noticeable symptoms in response to breast milk alone. The most common FPIES-specific symptoms noted in response to breast milk were diarrhea (34.1%), diarrhea with mucous or blood (58.5%), and vomiting (31.7%). Common nonspecific symptoms included acid reflux, changes in mood/temperament, rash, abdominal discomfort, gas, and increased night waking. Less commonly reported signs and symptoms were dehydration, lethargy, shock, constipation, and failure to thrive (Table 1). A more detailed breakdown of age of onset, symptoms, number of food triggers, and reactions to breastmilk between acute, chronic, and both acute and chronic infants can be found in Table 2.

**TABLE 2.** Comparison of acute, chronic, and both acute and chronic FPIES infants

Characteristics	No. of acute FPIES, % (n = 8)	No. of chronic FPIES, % (n = 10)	No. of both chronic and acute FPIES, % (n = 23)
Age of onset			
Birth	0	2 (20%)	3 (13%)
0–2 months	1 (12.5%)	7 (70%)	12 (52.2%)
3–4 months	1 (12.5%)	1 (10%)	1 (4.4%)
5–6 months	5 (62.5%)	0	5 (21.8%)
7–8 months	1 (12.5%)	0	1 (4.4%)
9–12 months	0	0	1 (4.4%)
Reaction symptoms			
FPIES-specific symptoms			
Vomiting	8 (100%)	6 (60%)	22 (95.6%)
Diarrhea with blood or mucous	2 (20%)	7 (70%)	20 (86.9%)
Diarrhea without blood or mucous	3 (38%)	8 (80%)	13 (56.5%)
Lethargy	8 (100%)	2 (20%)	19 (82.6%)
Weight loss	1 (12.5%)	4 (40%)	12 (52.2%)
Failure to thrive	0	2 (20%)	7 (30.4%)
Shock	2 (20%)	0	7 (30.4%)
Nonspecific symptoms			
Increased gas	1 (12.5%)	9 (90%)	15 (65.2%)
Acid reflux	2 (20%)	10 (100%)	20 (87%)
Abdominal pain	1 (12.5%)	9 (90%)	18 (78.3%)
Increased night waking	1 (12.5%)	9 (90%)	19 (82.6%)
Rash	2 (20%)	7(70%)	13 (56.5%)
Changes in mood/temperament	0	9 (90%)	21 (91.3%)
Constipation	1 (12.5%)	8 (80%)	9 (39.1%)
Dehydration	1 (12.5%)	2 (20%)	10 (43.4%)
No. of food triggers			
1	3 (32.5%)	0	0
2	3 (32.5%)	1 (10%)	0
3–4	2 (25%)	2 (20%)	9 (39.1%)
5-6	0	1 (10%)	2 (8.7%)
7+	0	6 (60%)	12 (52.2%)
Number reporting symptoms in response to to breastmilk	4 (50%)	8 (80%)	22 (96%)
Other allergic condition			
IgE-mediated food allergy	0	3 (30%)	4 (57.1%)
Atopic dermatitis	0	0	2 (28.6%)
Anaphylaxis	0	1 (10%)	1 (14.3%)

FPIES = food protein-induced enterocolitis syndrome.

The rate of formula feeding, breastfeeding, and combination (breastmilk and formula) feeding was similar between the FPIES and allergy-free infants (*P* = 0.95, 0.94, and 0.97, respectively). There was no difference in daycare attendance, pet ownership, C-section delivery rates, or gender between the two groups (Table 3).

**TABLE 3.** Self-reported demographic comparison between FPIES infants and allergy-free infants

	FPIES infants, % (n = 41)	Allergy-free infants, % (n = 34)	χ2, *P* value
Formula fed	5 (12.2%)	4 (11.8%)	0.95
Breastfed	25 (61.0%)	21 (61.8%)	0.94
Combination fed	11 (26.8%)	9 (26.5%)	0.97
C-section	8 (19.5%)	10 (29.4%)	0.32
Antibiotics prior to pregnancy	19 (46.3%)	14 (41.2%)	0.64
Prenatal antibiotics	20 (48.8%)	8 (23.5%)	0.024*
Antibiotics during delivery	17 (41.5%)	12 (35.3%)	0.58
Maternal antibiotics: breastfeeding	11 (26.8%)	6 (17.6%)	0.35
Infant antibiotic usage	18 (43.9%)	7 (20.6%)	0.033*
Families with a cat or dog	31 (75.6%)	21 (61.8%)	0.20
Day care attendance	4 (9.8%)	5 (14.3%)	0.54
Sex: male	28 (68.4%)	18 (52.3%)	0.16

FPIES = food protein-induced enterocolitis syndrome.

*Denotes a significant difference between the populations with *P* < 0.05.

Significant differences in antibiotic usage between groups were observed. Infant antibiotic usage was significantly higher in the FPIES group compared to the allergy-free infants (χ^2^ (1) = 4.54, *P* = 0.033, Table 3). χ^2^ analyses were used to tease out specifically when the use of antibiotics by the mother had the greatest influence on the probability of an infant acquiring FPIES. Three categories of antibiotic “timings” were analyzed: before pregnancy, during pregnancy, and during breastfeeding. The proportion of children with FPIES was not associated with maternal antibiotic use before pregnancy (χ^2^ (1) = 0.20, *P* = 0.65), as well as during breastfeeding (χ^2^ (1) = 0.89, *P* = 0.34). In contrast, antibiotic usage during pregnancy (prenatal usage) was associated with the occurrence of FPIES (χ^2^ (1) = 5.07, *P* = 0.024, Table 3). Since chronic FPIES was considered presumptive due to diagnostic criteria being poorly defined, χ^2^ analysis was repeated excluding participants who were reported to have just chronic FPIES. Prenatal antibiotic usage remained greater in all FPIES infants when compared to allergy-free infants (χ^2^ (1) = 8.058, *P* = 0.0045). Infant antibiotic usage also remained significantly greater in all FPIES infants when compared to allergy-free infants (χ^2^ (1) = 3.84, *P* = 0.050).

χ^2^ analyses were completed to examine other potential differences between those who were reported as acute FPIES compared to those who were reported as both acute and chronic FPIES. Age of symptom onset was grouped into 2 categories: 0 to 4 months and 5 to 12 months. Significantly more infants with both acute and chronic FPIES were diagnosed in the earlier 0- to 4-month age group compared to those with just acute FPIES (χ^2^ (1) = 4.84, *P* = 0.027, Table 4). Infants with both acute and chronic FPIES were more likely to report reactions to breast milk than infants with just acute FPIES (χ^2^ (1) = 9.14, *P* = 0.0025, Table 4). The number of food triggers were grouped into 2 categories: 1 to 4 triggers and 5+ triggers. Infants with both acute and chronic FPIES had significantly more food triggers when compared to those with just acute FPIES (χ^2^ (1) = 6.43, *P* = 0.011, Table 4). Caregivers of infants with both acute and chronic FPIES were more likely to report the following nonspecific symptoms when compared to infants with just acute: increased gas, acid reflux, abdominal pain, increased waking at night, and changes in mood (*P* < 0.05, Table 4). Additionally, infants with both acute and chronic FPIES were more likely to report diarrhea with blood or mucous when compared to just acute infants (χ^2^ (1) = 4.04, *P* = 0.044, Table 4). Prenatal antibiotic usage and infant antibiotic usage was significantly higher in those with both acute and chronic FPIES when compared to those with just acute FPIES (χ^2^ (1) = 10.26, *P* = 0.0014 and χ^2^ (1) = 3.84, *P* =0.05, respectively, Table 4).

**TABLE 4.** Summary of significantly different characteristics (*P* < 0.05) between acute FPIES infants and both acute and chronic FPIES infants

	Both acute+chronic FPIES, % (n = 23)	Acute FPIES, % (n = 8)	χ^2^, *P* value
Age of onset			
0–4 months	16 (69%)	2 (25%)	0.027
5–12 months	7 (30%)	6 (75%)	0.027
Reaction to breast milk	22 (96%)	4 (50%)	0.002
Prenatal antibiotics	17 (74%)	1 (12%)	0.001
Infants antibiotics	12 (52%)	1(12%)	0.050
No. of triggers			
1–4 foods	9 (39%)	8 (100%)	0.011
5+ foods	14 (61%)	0 (0%)	0.011
Nonspecific symptoms			
Increased gas	15 (65%)	1 (13%)	0.004
Reflux	20 (87%)	2 (20%)	0.044
Abdominal pain	18 (78%)	1 (13%)	0.021
Change in mood	21 (91.3%)	0 (0%)	0.003
Waking at night	19 (83%)	1 (13%)	0.014
FPIES-specific symptom			
Diarrhea with blood and mucous	20 (87%)	2 (20%)	0.044

FPIES = food protein-induced enterocolitis syndrome.

## DISCUSSION

Overall, our FPIES demographic had some similarities to past subsets of studied FPIES infants while other characteristics varied. The rate of coexistent IgE allergy (17%), while slightly higher in this study, was similar to other reported rates.^17,18^ Previous studies have typically noted more males with FPIES, and in the current study, there were approximately twice as many males (28) as females (13) with FPIES.^19–22^ We also found that most FPIES caregivers noticed symptoms at either 0 to 2 months or 5 to 6 months of age, which is in line with previous research that suggests symptom onset typically occurs earlier with a milk or soy-based formula introduction or later in infancy when solids are introduced.^23^ The majority of FPIES respondents reported multiple triggers, with 51% reporting 5 or more directly ingested food triggers. Most previous reporting describes acute FPIES patients with reactions to a single food.^17,24^ However, 2 recent pediatric cohorts in the United States also noted a significant number of participants with multiple food triggers. In the first pediatric cohort, 50% of the population had multiple food triggers,^6^ and in the second cohort, 45% had multiple triggers.^25^

While rates of C-section delivery and breastfeeding did not differ between the FPIES and allergy-free infants, there was significantly more antibiotic usage in FPIES infants and their mothers during pregnancy. Evidence suggests that the infant microbiome plays a role in IgE-mediated food allergy, asthma, and eczema, but this connection has not been explored with FPIES infants.^26^ Since maternal antibiotic usage during pregnancy has been associated with allergy, asthma, and eczema in infants, it is plausible that maternal antibiotic usage is associated with FPIES.^10,27–29^ The increase in maternal antibiotic usage seen in the FPIES group could indicate that the maternal microbiome impacts FPIES development. The maternal microbiome has a significant influence on the infant microbiome, and the maternal microbiome has been shown to play a critical role in the development of IgE-mediated food allergy and asthma.^9,11,30,31^ The relationship between antibiotic usage and FPIES seen in this study could also indicate that infection, resulting in the need for antibiotics, may be a factor in the development of FPIES. It is also possible that the mother’s immune system, which makes her more susceptible to infection, plays a role in the development of FPIES. This study did not obtain the type of infection that precipitated antibiotic usage nor the type of antibiotic utilized. Future research into FPIES and antibiotic usage should examine the type of antibiotic used, the type of infection warranting antibiotic usage, and the specific timing of antibiotic usage in both infants and pregnant mothers.

Recent evidence suggests that the innate immune system may play a critical role in the development of FPIES.^32,33^ In a population of patients with cow’s milk–induced FPIES, an increase in serum interleukin-8 and serum tryptase was noted, suggesting neutrophil and mast cell involvement.^32^ Whole blood profiling of a separate FPIES patient population demonstrated significant activation of eosinophils, monocytes, neutrophils, and natural killer cells following a supervised food challenge.^33^ While the role of the antibiotic usage in the development of FPIES is speculative, the host microbiome has been found to impact the innate immune system. Recently, a unique host protein called serum amyloid A suppressed neutrophil activation in response to the intestinal microbiota.^34^ While host microbes are known to interact with and activate mast cells, the relationship between the microbiome and mast cell activation in disease still needs to be elucidated.^35^ With the significant association between antibiotic usage and FPIES found in the current study, further study of the FPIES infant microbiome is recommended.

The survey data indicated significant differences between infants with just acute FPIES and infants who reported both acute and chronic FPIES. As previously mentioned, chronic FPIES must be considered presumptive due to the lack of well-defined clinical guidelines. However, there were clear differences between those who reported both and those who reported acute alone. Infants reported as both were more likely to react to breast milk, have more food triggers, and report onset of symptoms at a younger age (0–4 months). Caregivers of infants reporting both were also more likely to report nonspecific symptoms in addition to the FPIES-specific symptoms and have exposure to antibiotics. More work is needed to further elucidate differences between infants with acute FPIES and infants who reported both acute and chronic FPIES. Future research should closely examine these two different manifestations of FPIES. Is one a more severe form of FPIES, an additional syndrome in conjunction with acute FPIES (such as food protein-induced allergic proctocolitis), or a different syndrome altogether?

While early-onset FPIES in infants is typically described as chronic or acute FPIES in response to a cow’s milk–based or soy-based formula,^1,23^ our survey data suggest that another group should be considered. Eighty-eight percent of the FPIES caregivers reported noticeable symptoms in response to breast milk alone. There are limited reports of chronic FPIES reactions to breast milk in previous studies, but this phenomenon remains largely undescribed in the scientific literature.^20,36,37^ However, allergic reactions to food proteins via breast milk have been reported, as 60% of allergic proctocolitis cases occur in exclusively breastfed infants.^38^ In the current study, 28 of 41 FPIES infants reported FPIES-specific symptoms (such as vomiting, diarrhea, failure to thrive) in response to breastmilk and 6 reported only nonspecific symptoms (such as acid reflux, abdominal pain, changes in mood). While it is important to recognize that nonspecific symptoms in addition to FPIES symptoms were common in our study cohort, nonspecific symptoms alone could also foster overconcern for chronic FPIES, since many of these symptoms are very common in the general infant population. However, in exclusively breastfed infants, maternal diet via breast milk should be considered in evaluating the clinical symptoms of an infant. It is important for general pediatricians and allergists to understand and recognize this adverse reaction in a timely fashion to ensure the high quality of life for our young infants, as pediatricians are at the forefront in providing routine medical care to these infants.

A limitation of the study was the self-reported data and self-selected participants. The FPIES families were primarily recruited from an online FPIES support group, as well as through the FPIES Foundation’s social media outlets that could lead to selection bias. Members of an online support group may be more severe than the average FPIES family, potentially explaining the large number of FPIES infants with multiple triggers and other symptoms. However, there is a critical need to better understand the different manifestations of FPIES. Medical practitioners should be aware of these potentially more severe FPIES patients and the accompanying syndrome to aid in more rapid diagnosis and better management.

Patient-reported surveys help to share the experience of FPIES families and add to the body of knowledge that aids in characterizing this syndrome. The caregiver of an FPIES infant can offer critical insight into this allergic disease that lacks diagnostic testing. This study found that adverse reactions to breastmilk were more common than previously reported. Such recognition could allow for more rapid diagnosis and better management of FPIES infants and their families. Significant differences between infants reported to have acute FPIES alone and infants reported to have both acute and chronic FPIES establish the critical need to more closely examine these different subtypes of FPIES to better support and diagnose FPIES infants. Perhaps most importantly, the association between antibiotic usage and FPIES warrants further study to understand the etiology of FPIES and to identify potential risk factors associated with FPIES.

## ACKNOWLEDGMENTS

We thank all of the families that participated in this study and the following Keene State College students for their assistance with this project: Vince Scuderi, Jesse Bardis, Lauren Marple, Amber Walker, and Madalyn Carroll. We also greatly appreciate the efforts of Dr Wayne Shreffler, MassGeneral Hospital for Children, for his editorial assistance. All authors have met the four criteria of authorship established by International Committee of Medical Journal Editors. Specifically, each author was responsible for the following: J.B. contributed to the study design, Institutional Review Board proposal, participant recruitment, survey design and delivery, data analysis and interpretation, and manuscript preparation and revision. L.S. contributed to the data analysis and interpretation (she completed the majority of the statistical analysis), manuscript preparation, and revision. Q.Y. contributed to the study concept and design, clinical expertise, survey design, participant recruitment, data interpretation, and manuscript preparation and revision.
